# Tenuigenin regulates succinylation via SIRT5 for suppressing the tumorigenicity of hepatocellular carcinoma cells

**DOI:** 10.3389/fphar.2026.1756680

**Published:** 2026-04-24

**Authors:** Yifan Yan, Chang Liu, Jiao Luo, Lianhong Pan

**Affiliations:** 1 Chongqing Key Laboratory of Development and Utilization of Genuine Medicinal Materials in Three Gorges Reservoir Area, Chongqing Engineering Research Center of Antitumor Natural Drugs, Chongqing Three Gorges Medical College, Chongqing, China; 2 Oral Center, Chongqing University Three Gorges Hospital, Chongqing, China

**Keywords:** hepatocellular carcinoma, SIRT5, succinylation, tenuigenin, tumorigenicity

## Abstract

**Introduction:**

Tenuigenin (TEN) is an active component extracted from the *Polygala tenuifolia* root and has been reported to show anti-insomnia, anti- amnesia, neuroprotective, and anti-inflammatory effects. In this study, we aimed to investigate the anti-tumor effects of TEN on hepatocellular carcinoma (HCC) cells and to explore any underlying molecular mechanisms.

**Methods:**

The anti-tumor effect of TEN was assessed on Hep3B and HCCLM3 by CCK8, qRT-PCR, flow cytometry, RNA-sequence (RNA-seq) and LC-MS *in vitro*. Tumor xenograft mode was performed to examine the anti-tumor properties of TEN *in vivo*.

**Results:**

Our findings demonstrated that TEN has strong anti-tumor effects in vivo and *in vitro*. Furthermore, TEN significantly inhibited HCC cell proliferation by inhibiting S-phase cell cycle entry, in addition to migration and promoted apoptosis in Hep3B and HCCLM3 cells. *In vivo*, TEN significantly suppressed tumor growth by decreasing Ki67 and BCL2 expression. Mechanistic exploration found that interference with central carbon metabolism via succinate metabolite synthesis inhibition was a mediator of these changes within HCC cells, suggesting that cancer cell metabolic pathways were affected by TEN treatment. In addition, TEN significantly increased the expression of desuccinylase SIRT5, leading to reduced global succinylation and specific histone H3 lysine 122 succinylation (H3K122su) while simultaneously modulating the ERK/JNK signaling pathway in HCC cells.

**Conclusion:**

TEN exerts its anti-tumor effect in HCC cells by targeting SIRT5-succinylation. Therefore, TEN is a strong potential candidate anti-tumor drug for targeted HCC treatment.

## Introduction

Hepatocellular carcinoma (HCC) is the sixth-most common cancer worldwide and is associated with a high mortality rate (B et al., 2018). HCC is highly insidious and often diagnosed at an advanced stage, resulting in an unsatisfactory 5-year patient survival rate of approximately 18% and lower (Wu et al., 2021; Long et al., 2019). The most commonly used clinical approaches for HCC include surgery, trans-arterial chemoembolization, and radiofrequency ablation (Buskaran et al., 2021). However, the underlying pathogenesis of HCC remains unclear. Some studies have reported that the progression of HCC is dependent on cellular processes, including necroptosis, the cell cycle, and metabolism (Pan et al., 2021; Hernández-Alvarez and Zorzano, 2021). Therefore, targeting these pathogenetic processes could represent a new direction for HCC treatment. Currently, targeted therapies available for the treatment of HCC include lenvatinib, sorafenib and cisplatin; however, these are often associated with side effects and drug resistance, which limits their therapeutic effect (Feng et al., 2021). Therefore, there is an urgent unmet clinical need to identify novel targets and develop targeted drugs to address the challenges in the treatment of HCC.

Normally, the liver is responsible for many essential metabolic pathways of nutrients in the body. The occurrence and development of the HCC tumor are known to involve various metabolic pathways, including the glycolytic pathway, amino acid metabolism pathway, and lipid metabolism pathway (Li et al., 2022). Moreover, tumor cell growth requires numerous nutrients to support the demands of oncogenic proliferation and metastasis; as such, cancer cells make a variety of metabolic adaptations to support these processes (Pavlova et al., 2022). The disease course of HCC is related to complex metabolic reprogramming and inflammation (Alannan et al., 2020).

Metabolic reprogramming has been closely linked to various protein post-translational modifications (PTMs) (Iozzo et al., 2025). Lysine succinylation is a PTM that can regulate protein stability and function (Liu et al., 2023). In recent years, the role of lysine succinylation in cancer has been extensively studied (Shi et al., 2023), and the enzymatic and non-enzymatic mechanisms regulating post-succinylated protein modifications have been described in multiple studies (Zhang et al., 2025). However, clarifying the regulatory role(s) and underlying mechanism(s) associated with succinylation in oncogenesis still requires further in-depth study to support novel tumor treatment approaches. In addition, some studies have shown that targeting abnormally regulated lysine succinylation in liver cancer can serve as a potential anti-cancer strategy (Wu et al., 2025), using inhibitors that target the regulatory effects of lysine succinylation modifications and elucidate the associated mechanisms. At present, studies on natural compounds that directly modulate succinylation are extremely limited.

Tenuigenin (TEN) is a natural product extracted from the roots of *Polygala tenuifolia*, and it has been shown to possess various pharmacological properties, including treatment of insomnia, improvement of cognitive function, and anti-inflammatory effects (Deng et al., 2020). For example, Zhang et al. (2023) found that TEN can ameliorate sleep disturbances in 1-methyl-4-phenyl-1,2,3,6-tetrahydropyridine (MPTP) -induced mice; another study reported that TEN was involved in the regulation of hippocampal neural stem cells, in addition to their proliferation and differentiation (Chen et al., 2012), and TEN was found to promote functional recovery in rats with spinal cord injury (SCI) through modulation of the IRS1/AKT/mTOR signaling pathway (Zang et al., 2023). Moreover, TEN also demonstrated strong anti-inflammatory activity by reducing NLRP3 inflammasome expression and downregulating the MAPK/NF-κB pathway (Fan et al., 2017; Lv et al., 2016). However, there have been no reports on whether TEN has any regulatory effects on or within HCC cells. In addition, given the extensive applications of metabolomics in drug and tumor research, it has not been reported whether TEN regulates the progression of liver cancer through its regulation of metabolic pathways driving HCC cells, which is worthy of further study.

Our study observed that TEN significantly inhibited the growth of HCC tumors in nude mice *in vivo*. Furthermore, TEN inhibited the proliferation and migration of HCC cells while promoting their apoptosis *in vitro* by altering cell-intrinsic metabolic pathways through regulation of succinylation modification via changes in succinate expression. TEN was also found to modulate cell proliferation and migration by significantly increasing the expression of the desuccinylase SIRT5, further decreasing succinylation while simultaneously inhibiting the ERK/JNK signaling pathway in HCC cells. Therefore, TEN is a strong potential drug candidate for the treatment of HCC due to its anti-tumor effects, which are attributed to the observed metabolism-mediated promotion of apoptosis and inhibition of proliferation in HCC cells.

## Materials and methods

### Cell culture

The liver cancer cell lines Hep3B and HCCLM3 were maintained using high-glucose Dulbecco’s modified Eagle’s medium (DMEM; Gibco, United States), containing 10% fetal bovine serum (FBS; Gibco, United States) and 1% penicillin–streptomycin (P/S; Gibco, United States). Cells were cultured in a humidified atmosphere with 5% CO_2_ at 37 °C.

### Drug treatment

TEN (molecular formula: C30H45ClO6; relative molecular mass: 537.13; purity ≥99.24%) was purchased from MedChemExpress Company (MCE, United States), dissolved in DMSO, and different concentrations (10, 20, and 50 μM) were used for the treatment of HCC cells. Cell morphology was imaged using an Olympus microscope (Tokyo, Japan).

### Cell viability assay

Hep3B and HCCLM3 cell viability was measured using a CCK-8 assay (Dojindo, Japan) as follows. First, cells were seeded into a 96-well plate (5,000 cells/well) and treated with TEN (10, 20, and 50 μM) for 48 h, in addition to cisplatin (CDDP) treatment (2 μg/mL). Next, CCK-8 (10 µL/well) was added, and cells were incubated in the dark at 37 °C for 2 h. Then, the absorbance of the cells at 450 nm was measured and recorded. The cell death rate was calculated as described in previous studies (Pan et al., 2022).

### Colony formation experiment

Cells were seeded into a 6-well plate at 1,000 cells/well and cultured in an incubator for 5 days. Cells were treated with TEN (10, 20, and 50 μM) or CDDP (2 μg/mL) as a positive control group. After 10 days of culture, cells were washed with PBS, fixed with 4% PFA, stained with 1% crystal violet, and photographed, after which the cell colonies were counted.

### Flow cytometry

Cells were treated with DMSO, TEN (10, 20, and 50 μM), or CDDP (2 μg/mL) as a positive control group for 48 h. For the cell cycle assay, cells were fixed with pre-chilled 75% ethanol for 12 h at 4 °C. Then, cells were washed three times with PBS and incubated with RNase A (Sigma Aldrich, United States) and propidium iodide (PI, BD, United States) at a 1:9 ratio in the dark for 1 h. For the apoptosis assay, cells were washed three times with PBS and re-suspended in 100 μL binding buffer (BD, United States) mixed with 5 μL FITC-labeled Annexin V (NeoBioscience, Beijing) and 2 μL PI (BD, United States) at room temperature (RT) in the dark for 20 min. Cells were analyzed using a DxFLEX flow cytometer (Beckman, United States).

For the reactive oxygen species (ROS) detection assay, a ROS detection kit (Beyotime, China) was used. Cells were treated with DMSO or TEN (20 or 50 μM) for 48 h, collected, and re-suspended in diluted DCFH-DA solution. Cells were incubated in the dark for 0.5 h, during which the tubes were agitated every 10 min to ensure maximum probe binding. Then, cells were analyzed using a DxFLEX flow cytometer (Beckman, Germany) at the 488 nm excitation wavelength.

### Western blot analysis

Cells were treated with DMSO, TEN (10, 20, and 50 μM), or CDDP (2 μg/mL) as a positive control group for 48 h. Then, the cells were collected and lysed using RIPA lysis buffer (Beyotime, China). In addition, samples were processed in a randomized order to minimize batch effects. The lysate protein concentration was determined using a BCA protein quantification kit (Beyotime, China), and the protein was denatured at 100 °C for 10 min before protein lysates were subjected to 10% SDS–PAGE gel electrophoresis and subsequent transfer of the separated proteins onto PVDF membranes (Millipore, United States). Subsequently, the PVDF membranes were blocked with 5% milk for 2 h and then incubated with primary antibodies against CDK2, CDK4, cyclin D1, N-cadherin, E-cadherin, vimentin, caspase 8, MCL1, survivin, or β-actin (purchased from ZENBIO) at 4 °C overnight. Then, the membranes were incubated with HRP-conjugated secondary antibodies for 2 h at RT, after which an ECL system (ZENBIO, China) was used to visualize membrane proteins. Protein quantities were normalized to β-actin.

### Cell migration assay

For the scratch assay, Hep3B and HCCLM3 cells were seeded into a 6-well plate. After reaching a density of 70%, cells were scratched using a sterile pipette and washed with pre-cold PBS. Then, TEN (10, 20, or 50 μM) or CDDP (2 μg/mL; as a positive control group) was added for 24 h. After that, the cells were fixed in the plates using 4% PFA and then stained with 0.1% crystal violet. After washing with PBS, microscopy images were taken.

For the Transwell assay, Hep3B and HCCLM3 cells were seeded into an 8-μm 24-well Transwell cell plate (Millipore, United States) at a density of 1× 10^4^ cells/well in the upper chamber with 2% FBS medium, while 15% FBS medium in the lower chamber was used as a chemoattractant. TEN (10, 20, or 50 μM) or CDDP (2 μg/mL; as a positive control) was added to the plates for 24 h. Following this, the cells were fixed with 4% PFA and stained with 0.1% crystal violet. After washing with PBS, microscopy images were taken.

#### TUNEL assay

HCCLM3 and Hep3B cells were seeded into a 48-well plate and treated with TEN (10, 20, and 50 μM) for 48 h before washing with PBS and cell fixation using 4% PFA at 37 °C for 0.5 h. Cells were then permeabilized with 0.1% Triton X-100. A TUNEL reagent (Mingbo, China) was added to each well according to the manufacturer’s protocol. Finally, DAPI was added and incubated for 0.5 h at RT to stain cell nuclei before the cells were washed with PBS and photographed using a microscope (Olympus, Japan).

#### EdU assay

HCCLM3 and Hep3B cells were seeded into a 96-well plate and treated with TEN (10, 20, or 50 μM) for 48 h before staining using an EdU detection kit (Beyotime, China) for the quantification of cell proliferation. In summary, cells were incubated with preheated EdU-labeling medium (10 μM) for 1.5 h for cell labeling and then fixed with 100 μL 4% PFA for 10 min and permeabilized with 100 μL permeation solution (Beyotime, China). Next, cells were treated with 50 μL of the mixed Click Additive Solution, after which Hoechst 33342 solution was added to stain the cell nuclei. Finally, the staining solution was removed, and the cells were washed and photographed using a microscope (Olympus, Japan).

#### RNA sequencing and qRT-PCR

After treatment with DMSO or TEN (20 μM) for 48 h, the cells were collected, and total RNA was isolated using TRIzol reagent (TIANGEN, China) and cryopreserved at −80 °C. Transcriptome sequencing (n = 3) was then performed by BGI Co., Ltd. (Shenzhen, China). Raw sequencing data were rigorously filtered and mapped to the human reference genome. Then, quantitative gene expression analysis was conducted, followed by various analyses, including differential expression and clustering. The relative expression of selected genes was measured by qRT-PCR using the ABI 7500 Instrument (ABI, United States). Differentially expressed genes (DEGs) (log_2_fold change ≥ 1 −log(*P*) ≥ 10) were included for downstream functional analysis. All primer information is listed in Table 1. Gene expression levels were normalized to β-actin.

**TABLE 1:** Primer sequences for real-time RT-PCR.

Gene	Primer sequence (forward/reverse)
CDK2 CDK4 CDK6 Cyclin D1 MCL1 BAX BCL2 Survivin Caspase 8 N-cadherin Vimentin MMP2 E-cadherin β-actin	5′-CCAGGAGTTACTTCTATGCCTGA -3′ 5′- TTCATCCAGGGGAGGTACAAC-3′ 5′- TCAGCACAGTTCGTGAGGTG-3′ 5′- GTCCATCAGCCGGACAACAT-3′ 5′-TCTTCATTCACACCGAGTAGTGC -3′ 5′-TGAGGTTAGAGCCATCTGGAAA -3′ 5′-GCTGCGAAGTGGAAACCATC -3′ 5′- CCTCCTTCTGCACACATTTGAA-3′ 5′-GTAATAACACCAGTACGGACGG-3′ 5′-CCACAAACCCATCCTTGGAAG-3′ 5′- CCTTTTCTACTTTGCCAGCAAAC -3′ 5′- GAGGCCGTCCCAACCAC -3′ 5′- GGTGGGGTCATGTGTGTGG -3′ 5′- CGGTTCAGGTACTCAGTCATCC -3′ 5′- AGGACCACCGCATCTCTACAT-3′ 5′- AAGTCTGGCTCGTTCTCAGTG-3′ 5′- GGATGATGACATGAACCTGCTGGA -3′ 5′- TTGTTGATTTGGGCACAGACTCTT -3′ 5′- AGCCAACCTTAACTGAGGAGT -3′ 5′- GGCAAGTTGATTGGAGGGATG -3′ 5′- AGTCCACTGAGTACCGGAGAC -3′ 5′- CATTTCACGCATCTGGCGTTC -3′ 5′- TACAGGATCATTGGCTACACACC -3′ 5′- GGTCACATCGCTCCAGACT-3′ 5′-CGAGAGCTACACGTTCACGG-3′ 5′-GGGTGTCGAGGGAAAAATAGG-3′ 5′-GGGAAATCGTGCGTGACATT-3′ 5′-TGCCCAGGAAGGAAGGCT-3′

### Liquid chromatography–mass spectrometry (LC–MS) experimental methodology

HCCLM3 cells were seeded in a 6-well plate and treated with 20 µM TEN for 48 h; then, the cells were washed three times with pre-cold PBS. Next, cell metabolites were extracted using 1 mL ice-cold lysis buffer (methanol: acetonitrile: water with 0.5% formic acid at a 40:40:20 ratio) per well, followed by a 5-min incubation on ice. Following this, 50 μL of 15% NH_4_HCO_3_ was added per well to neutralize the superfluous acetic acid. Cells were scraped and collected in a sterilized EP tube, followed by centrifugation. As previously described, samples (n = 6) were processed in a randomized order to minimize batch effects. Next, the metabolite-containing supernatant was transferred to a fresh and sterilized EP tube for cryopreservation at −80 °C. BGI Co., Ltd. (Shenzhen, China) conducted LC–MS sequencing and analysis. Metabolomic data were log_10_-transformed and analyzed using Progenesis QI software for peak detection, extraction, alignment, and integration. Metabolites were further annotated against public databases. Metabolites with variable importance in the projection (VIP) > 1, fold change (FC) > 1.5, and *p* < 0.05 were considered differentially abundant between groups.

### RNA interference assay

A small interfering RNA (SiRNA) sequence targeting SIRT5 was designed and synthesized by RiboBio (Guangzhou, China). Cultured cells were transfected using Lipofectamine 3000 (Thermo Fisher, United States), following the manufacturer’s protocol. Then, the protein level of SIRT5 was validated using Western blotting. The siRNA oligo sequences are as follows: 5′-CAGCAUCCCAGUUGAGAAAdTdT-3’.

### Plasmid construction and transfection assay

Cells were transfected with the overexpression vector (oe-SIRT5) using the Lipofectamine 3000 reagent (Thermo Fisher, United States), after which the cells were cultivated for 24 h. Subsequently, the cells were collected for protein lysis, and the protein level of SIRT5 was quantified using Western blotting, followed by further functional testing.

### *In vivo* anti-tumor study and histochemistry analysis

Female nude mice (∼4-weeks-old) were purchased from Beijing Weitong Lihua Laboratory Animal Technology Co., Ltd. Hep3B cells (1 × 10^6^) in 100 μL of Matrigel were injected into the right axillary region of each mouse (Corning, United States). Mice were then fed for 15 days, and the volumes were measured. At the experimental point when the longest axis of the tumor was between 5 and 8 mm, mice were randomly divided into five groups (n = 6 each). All animal experiments were performed with randomization and blinding. Mice were randomly assigned to treatment groups using a random number generator. TEN (5, 10, 15, or 20 mg/kg, dissolved in the vehicle solution) or the vehicle solution only was injected intraperitoneally daily. Tumor volumes were measured and calculated as follows: (π/6) × length × width^2^. Tumor measurements and endpoint analyses were conducted by investigators blinded to group allocation. After mice were injected with TEN for seven consecutive doses, they were euthanized using a gradually increasing concentration of CO_2_ (at a flow rate of 30%–70% of the container volume per minute), after which death was confirmed by cervical dislocation. Finally, the tumors and major organs (heart, liver, spleen, lungs, and kidneys) were collected, weighed, and fixed using 4% PFA. Tissues were paraffin-embedded and sectioned to perform H&E staining, immunohistochemical (IHC) staining, and immunofluorescence (IF) staining. For IHC quantification, all slides were coded and scored independently by two pathologists blinded to the experimental groups. All animal experiments were authorized and supervised by the Ethics Committees of Chongqing Three Gorges Medical College (protocol code SXYZ-A-202306-0004).

### Statistical analysis

All experiments were repeated with ≥3 technical and biological replicates, and data are presented as the mean ± SD. One‐way ANOVA was used for multiple comparisons, whereas *t* tests were used for two‐group comparisons. Group differences within the experimental data were considered statistically significant at *p* < 0.05 and extremely statistically significant at *p* < 0.01.

## Results

### TEN inhibits the viability of Hep3B and HCCLM3 cells

**FIGURE 1:**
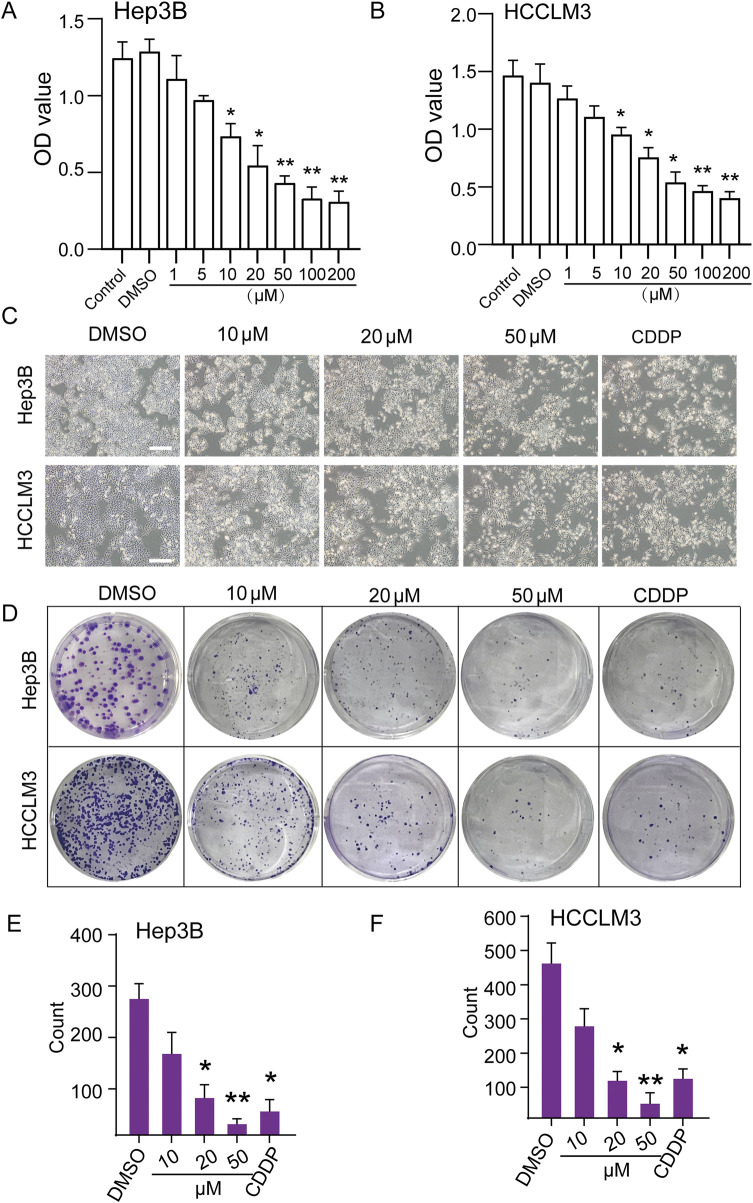
TEN inhibits HCC cell viability and proliferation in a concentration-dependent manner. **(A,B)** Cell viability of Hep3B and HCCLM3 cells treated with TEN at the indicated concentration was determined using the CCK-8 method. DMSO was used as the control. n = 6. **(C)** The morphology of Hep3B and HCCLM3 cells treated with TEN at different concentrations of 10, 20, and 50 μM for 48 h. DMSO was used as the control, and 2 μg/mL CDPP was used as the positive control. Scale bar = 100 µm. **(D‐F)** Clone formation of Hep3B and HCCLM3 cells treated with TEN at 10, 20, and 50 μM. DMSO was used as the control, and 2 μg/mL CDDP was used as the positive control. n = 5. One‐way analysis of variance (ANOVA) was employed for multiple comparisons, whereas *t* tests were used for two‐group comparisons. *p < 0.05 and * *p < 0.01 indicate significant differences compared with the control group.

Hep3B and HCCLM3 cells were treated with different concentrations of TEN for 48 h and assayed using a CCK8 assay, which showed that TEN significantly inhibited the growth and viability of Hep3B and HCCLM3 cells (Figures 1A,B). Next, significant morphological changes were observed following TEN treatment; as the concentration increased, the proportion of adherent cells decreased proportionately, while detached cells increased significantly (Figure 1C). Furthermore, both TEN and CDDP treatment resulted in a significant reduction in the number of HCC cell colonies, and the number of colonies in the 50 μM TEN treatment group was less than that in the CDDP group (Figures 1D–F). These findings suggested that TEN inhibited the cell growth and viability of Hep3B and HCCLM3 cells.

### TEN regulates genes expression in HCCLM3 cells

**FIGURE 2:**
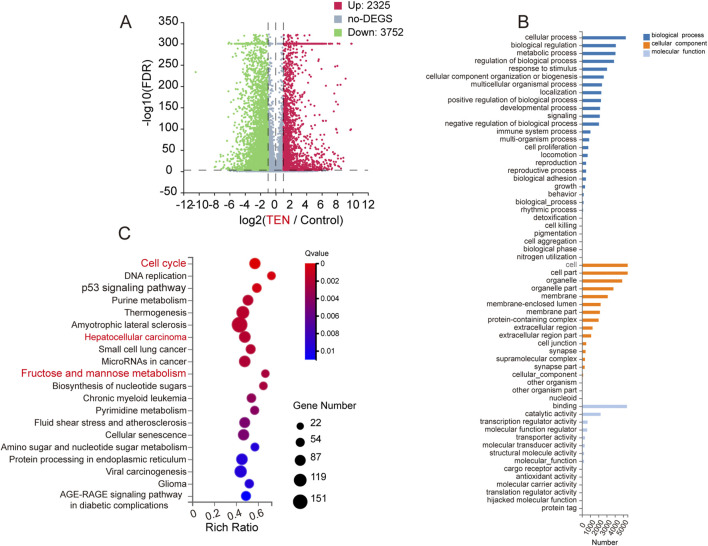
TEN regulates the differentially expressed genes and pathway enrichment analysis in HCCLM3 cells. **(A)** Significant differentially expressed genes are shown in the volcano plot. FC (fold change) > 1 was accepted as positive differentially expressed genes, up for 2,325; down for 3,752. **(B)** GO annotation analysis of HCCLM3 cells treated with TEN (20 μM), compared with the control group. **(C)** KEGG pathway enrichment analysis, a larger *p*-value (−Log10) indicates a higher degree of enrichment. Significant pathways involving in metabolic pathways of HCCLM3 cells treated with TEN. n = 3.

Transcriptomics analyses revealed that TEN treatment significantly deregulated a wide range of gene expression in HCCLM3 cells. Following TEN (20 μM) treatment in HCCLM3 cells, 6,077 DEGs were observed: 2,325 genes were upregulated (in red), and 3,752 genes were downregulated (in green), indicating that TEN significantly deregulates gene transcription, thereby controlling basic cellular functions (Figure 2A). GO annotation analysis showed that the DEGs were highly enriched in a number of biological process classifications, including the pathways classified as cellular processes, biological regulation, and metabolic processes (Figure 2B). In addition, KEGG enrichment analysis indicated that the cell cycle pathway and the fructose and mannose metabolism pathway were significantly enriched in the DEGs following TEN treatment, suggesting that TEN may regulate the biological functions and cell fate of HCC cells through these pathways (Figure 2C).

### TEN inhibits the S-phase cell cycle in Hep3B and HCCLM3 cells

**FIGURE 3:**
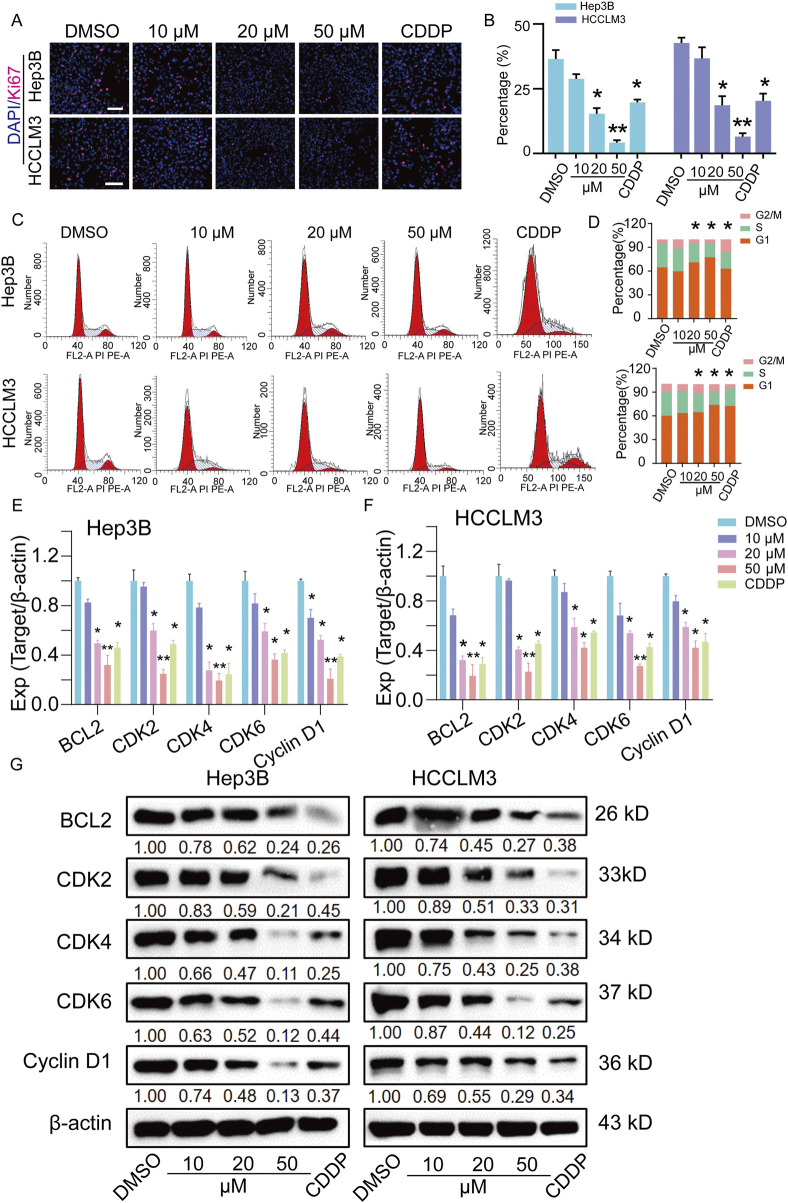
TEN inhibits the cell proliferation of HCC cells. **(A,B)** Cell proliferation from Ki67 staining assay of HCC cells following treatment with TEN for 48 h. n = 5. **(C,D)** Cell cycles of Hep3B and HCCLM3 cells treated with TEN at 10, 20, and 50 μM for 48 h were analyzed using flow cytometry. n = 4. **(E,F)** The gene expressions of BCL2, CDK2, CDK4, CDK6, and cyclin D1 in Hep3B and HCCLM3 cells treated with TEN at 10, 20, and 50 μM for 48 h. The relative expression levels were analyzed using the 2^−ΔΔct^ method. n = 4. **(G)** The representative bands of BCL2, CDK2, CDK4, CDK6, and cyclin D1 in Hep3B and HCCLM3 cells treated with TEN at 10, 20, and 50 μM for 48 h. β-actin was used as an internal control. n = 3. One‐way analysis of variance (ANOVA) was employed for multiple comparisons, whereas *t* tests were used for two‐group comparisons. *p < 0.05 and * *p < 0.01 indicate significant differences compared with the control group.

Next, to evaluate the effect of TEN on the proliferation of HCC cells, a Ki67 staining assay revealed that Ki67^+^ cells were reduced after TEN treatment, indicating that cell proliferation was inhibited by TEN (Figures 3A,B). This aligns with the KEGG analysis showing that the transcriptional changes were highly enriched in the cell cycle. Validation using flow cytometry showed that TEN treatment inhibited cell cycle entry into the S-phase: TEN treatment (50 μM) reduced the proportion of S-phase cells in Hep3B cells (9.2%) and HCCLM3 cells (11.43%) (Figures 3C,D). Furthermore, using qRT-PCR and Western blotting, TEN decreased the expressions of BCL2, CDK2, CDK4, CDK6, and cyclin D1 at both gene and protein levels, and this effect was significantly stronger in the 50 μM TEN treatment group than in the CDDP treatment group (Figures 3E–G). Together, these results confirmed that TEN can regulate cell proliferation by influencing the transition through the G1/S phases of the cell cycle, providing strong evidence for an anti-HCC effect of TEN *in vitro*.

### TEN induces the apoptosis of Hep3B and HCCLM3 cells

**FIGURE 4:**
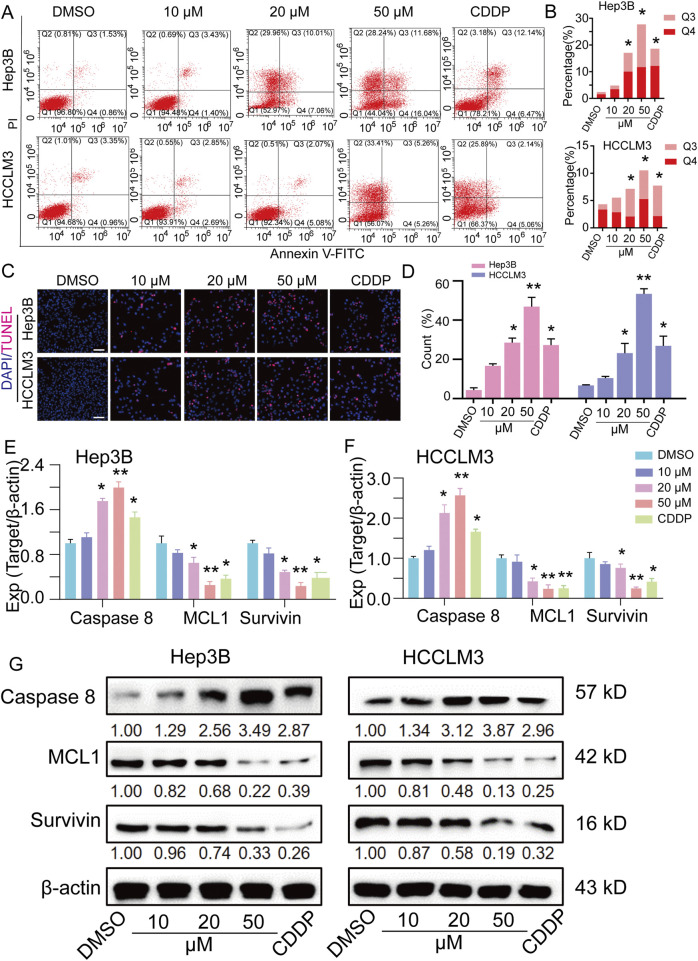
TEN induces apoptosis in HCC cells. **(A,B)** Apoptosis of Hep3B and HCCLM3 cells treated with TEN at 10, 20, and 50 μM for 48 h was analyzed using flow cytometry. n = 4. **(C,D)** Cell apoptosis from the TUNEL assay of HCC cells following treatment with TEN for 48 h. n = 5. **(E,F)** The gene expression of caspase 8, MCL1, and survivin in Hep3B and HCCLM3 cells treated with TEN at 10, 20, and 50 μM for 48 h. The relative expression levels were analyzed using the 2^−ΔΔct^ method. n = 4. **(G)** The representative bands of BAX, BCL2, MCL1, and caspase 8 in Hep3B and HCCLM3 cells treated with TEN at 10, 20, and 50 μM for 48 h. n = 3. β-actin was used as an internal control. ANOVA was employed for multiple comparisons, whereas *t* tests were used for two‐group comparisons. *p < 0.05 and * *p < 0.01 indicate significant differences compared with the control group.

Using flow cytometry assays, we further investigated the effect of TEN on the apoptosis of liver cancer cells, finding that both Hep3B and HCCLM3 cells exhibited an increase in apoptosis following 48 h of TEN treatment: in Hep3B cells, apoptosis was increased by 2.44%, 14.68%, and 25.33% in the 10 μM, 20 μM, and 50 µM groups, respectively, whereas in HCCLM3 cells, apoptosis rates were increased by 1.23%, 3.52%, and 6.25%, respectively. In addition, the apoptosis rate of cells in the 50 µM group was higher than that in the CDDP group (Figures 4A,B). These results indicate that TEN induces apoptosis in HCC cells. Next, TUNEL staining showed that TEN treatment significantly increased the number of apoptotic cells (Figures 4C,D). Furthermore, using qRT-PCR and Western blotting, TEN treatment was shown to reduce the expression of MCL1 and survivin, while caspase 8 expression was induced at both the gene and protein levels. Additionally, these changes were significantly greater in cells treated with 50 μM TEN than in those treated with CDDP (Figures 4E–G). These results suggest that TEN promotes programmed cell death by regulating the expression of cell apoptosis-related genes and proteins in HCC cells.

### TEN suppressed cell migration in Hep3B and HCCLM3 cells

**FIGURE 5:**
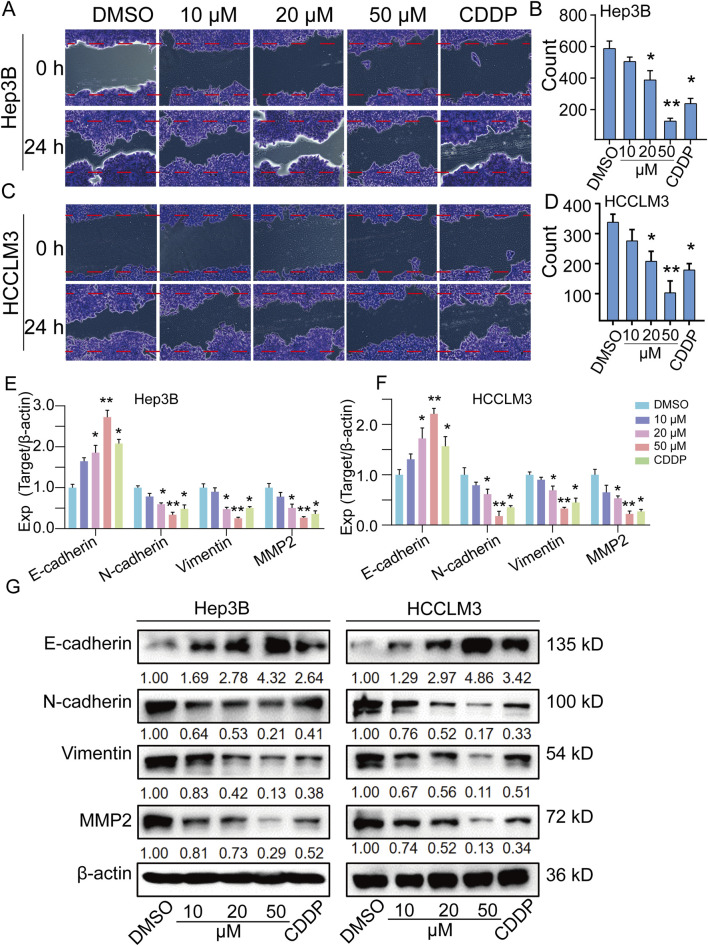
TEN inhibits cell migration in HCC cells. **(A–D)** Cell scratch assays of Hep3B and HCCLM3 cells treated with TEN at 10, 20, and 50 μM for 24 h n = 5. **(E,F)** Gene expression of E-cadherin, N-cadherin, vimentin, and MMP2 in Hep3B and HCCLM3 cells treated with TEN at 10, 20, and 50 μM for 24 h. The relative expression levels were analyzed using the 2^−ΔΔct^ method. n = 4. **(G)** The representative bands of E-cadherin, N-cadherin, vimentin, and MMP2 in Hep3B and HCCLM3 cell treated with TEN at 10, 20, and 50 μM for 24 h. β-actin was used as an internal control. n = 3. One‐way analysis of variance (ANOVA) was employed for multiple comparisons, whereas *t* tests were used for two‐group comparisons. *p < 0.05 and * *p < 0.01 indicate significant differences compared with the control group.

Subsequently, a scratch assay was conducted to assess the effects of TEN on HCC cell migration. Our results indicate that TEN treatment significantly inhibited the migration of HCC cells at various concentrations, and the inhibitory effect observed in the 50 µM TEN treatment group was stronger than that in the CDDP group (Figures 5A–D). Furthermore, qRT-PCR analysis and Western blot analysis both confirmed that the expression of migration-related genes and proteins was significantly deregulated after treatment. Specifically, the expressions of migration-related proteins N-cadherin, vimentin, and MMP2 were inhibited, while the expression of migration-related protein E-cadherin was promoted (Figures 5E–G). Collectively, these changes indicate that TEN can effectively regulate the migration activity of HCC cells *in vitro*.

### TEN treatment reprograms carbon metabolism in HCCLM3 cells

Malignant tumorigenesis is often accompanied by abnormal cellular metabolism, which is a key component in the mechanistic development of tumors. From the transcriptomics analyses, metabolic processes were significantly enriched in the TEN-associated DEGs, whereby KEGG analysis highlighted the enrichment of the fructose and mannose metabolism pathways. These are important processes in glucose metabolism, so we used an LC–MS metabolomic approach for further validation in HCCLM3 cells to assess the effect of TEN treatment on the metabolic products of HCC cells.

**FIGURE 6:**
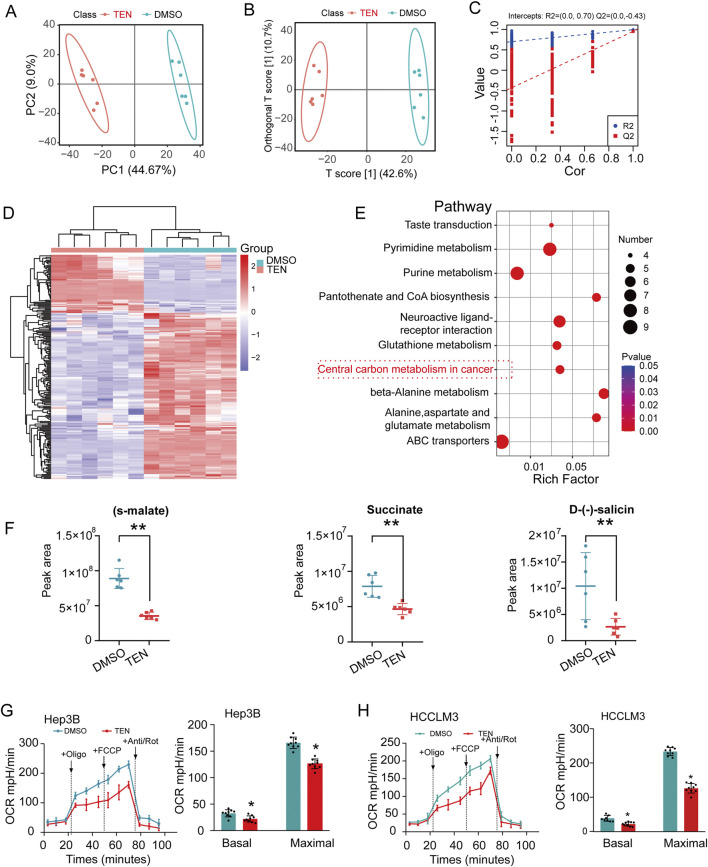
TEN regulates the central carbon metabolism in the cancer pathway in HCCLM3 cells. **(A)** The PCA score plot of the control and TEN groups (20 μM); it represents that samples in the control and TEN groups were closely clustered to one another. n = 6. **(B)** The OPLS-DA score plot of control and TEN groups revealed the clustering of samples of control and TEN groups in the training set. **(C)** The OPLS-DA verification model of control and TEN groups. **(D)**Heatmap of metabolites showing between control and TEN groups. Increased metabolites were marked in red. Decreased metabolites levels were presented in blue. **(E)** Plots depict the computed metabolic pathways as a function of −log (p) (y-axis) and the pathway impact of the key metabolites (x-axis) that differed between the control and TEN groups. The size of a circle is proportional to the pathway impact value of the pathway. **(F)** Metabolites related to central carbon metabolism in the cancer pathway altered by TEN treatment in HCCLM3 cells. **(G,H)** Oxygen consumption rates (OCRs) of HCC cells were measured with or without TEN. n = 6 replicates per group. *p < 0.05 and * *p < 0.01 indicate significant differences compared with the control group.

First, principal component analysis (PCA) revealed that the vehicle control and 20 μM TEN treatment groups were tightly clustered (Figure 6A). OPLS-DA was then used to exclude interfering factors unrelated to the inter-group differences, and a statistical assessment of the relevant signals was conducted, showing that the differences within each group in the two sets of samples were relatively small (Figure 6B). The OPLS-DA validation model is an external validation of OPLS-DA designed to avoid over-fitting; the data indicated that the OPLS-DA model between the control and drug groups showed a good fit and clear differences (Figure 6C). Next, the heat map shown in Figure 6D indicates that TEN treatment could affect the production of various primary metabolites in HCC cells. In addition, a KEGG enrichment analysis indicated that central carbon metabolism in the cancer pathway was significantly enriched in the deregulated metabolites, suggesting a potential reprogramming of central carbon metabolism (Figure 6E). In addition, differential metabolite analysis revealed that TEN treatment significantly inhibited the expression of s-malate, succinate, and D-(−)-salicin (Figure 6F). Furthermore, intracellular succinate content analysis indicated that TEN significantly reduced the level of succinate within HCCLM3 and Hep3B cells (Supplementary Figure S1). The tricarboxylic acid cycle (TCA) is the core of the central carbon metabolism in the cancer pathway and is known to be deregulated in cancer cells. To further verify the effect of TEN treatment on the TCA cycle in HCC cells, we used the Seahorse XF extracellular flux analyzer and found that TEN treatment significantly inhibited oxidative phosphorylation in HCC cells, indicating that TEN can alter the TCA cycle and reprogram central carbon metabolism in HCC cells to affect their biological behaviors (Figures 6G,H). Together, these data suggest that TEN treatment could exert anticancer effects by regulating the central carbon metabolism.

### TEN demonstrated anti-HCC effects by regulating succinylation and targeting the ERK signaling pathway

**FIGURE 7:**
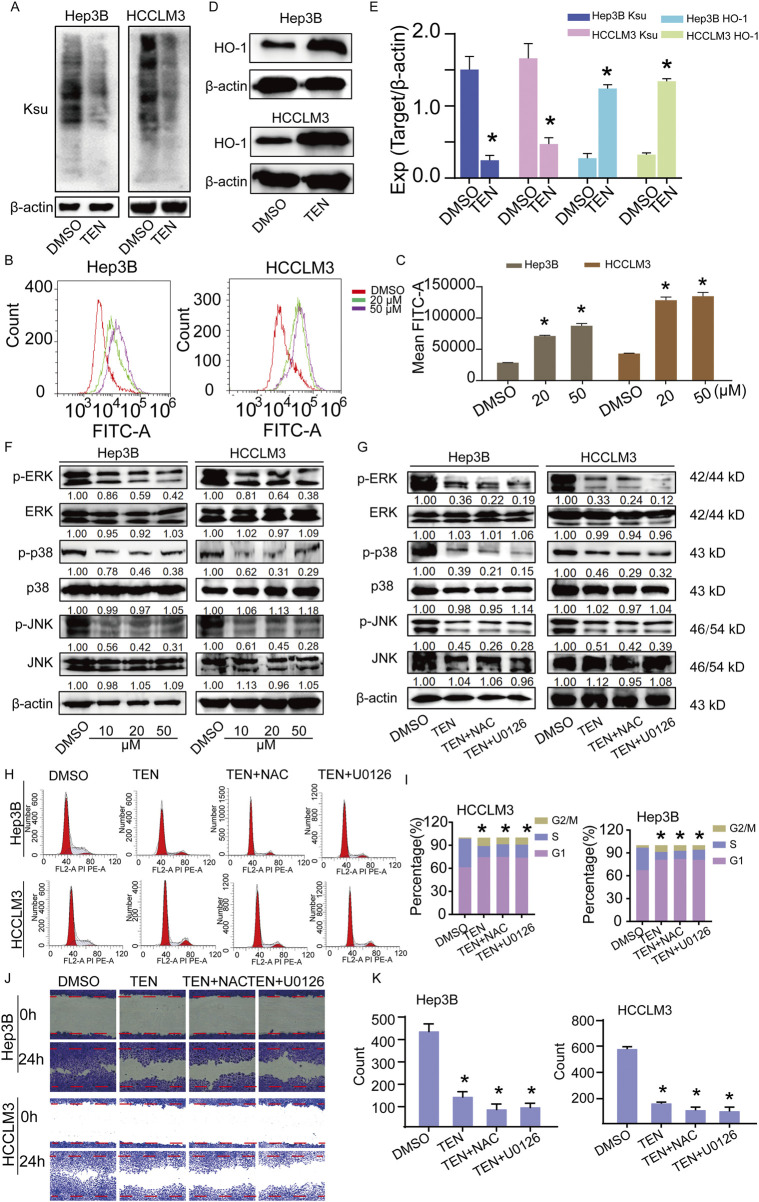
TEN inhibits the biological behavior of HCC cells by regulating succinylation and targeting the ERK signaling pathway. **(A)** The protein expression of Ksu in Hep3B and HCCLM3 cells treated with TEN (20 μM) for 48 h. n = 3. **(B,C)** The expression of ROS in Hep3B and HCCLM3 cells treated with TEN (20 μM) for 48 h. n = 5. **(D)** The protein expression of HO-1 in Hep3B and HCCLM3 cells treated with TEN (20 μM) for 48 h. n = 3. **(E)** Quantitative image analysis of **(A,D)**. **(F,G)** Western blotting analysis for detecting p-ERK and p-p38, p-JNK at the protein level with or without the ROS inhibitor (NAC, 3 mM), and the ERK pathway inhibitor (U0126, 10 μM) in HCC cells. **(H,I)** Cell cycle analysis of Hep3B and HCCLM3 cells treated with TEN (20 μM), the ROS inhibitor (NAC, 3 mM) and the ERK pathway inhibitor (U0126, 10 μM). n = 3. **(J,K)** Cell migration analysis of Hep3B and HCCLM3 cells treated with TEN (20 μM), theROS inhibitor (NAC, 3 mM), and the ERK pathway inhibitor (U0126, 10 μM). n = 5. One‐way analysis of variance (ANOVA) was employed for multiple comparisons, whereas *t* tests were used for two‐group comparisons. *p < 0.05 indicates significant differences compared with the control group.

Since TEN treatment was shown to potentially reprogram the central carbon metabolism in the cancer metabolism pathway by down-regulating succinate expression in HCCLM3 cells, we further investigated the relationship between TEN treatment and succinylation. These experiments found that succinylation levels were reduced by TEN treatment in HCC cells (Figure 7A). In addition, our data unequivocally demonstrate that TEN regulates the succinylation modification of H3 at the H3K122su site in HCC cells (Supplementary Figure S2). Moreover, succinylation can lead to the production of ROS in tumor cells, which can functionally impact cell behavior (Chen et al., 2024). We evaluated this relationship using a DFCH-DA probe experiment, which showed that ROS levels in HCC cells significantly increased after TEN treatment (Figures 7B,C). Furthermore, HO-1 expression was significantly up-regulated following TEN treatment (Figures 7D,E). By investigating the underlying anti-tumorigenic mechanism of TEN treatment in HCC cells, it was demonstrated that TEN treatment led to significantly downregulated phosphorylation of ERK, p38, and JNK proteins (Figure 7F). Treatment with the ROS inhibitor NAC (3 mM) or the ERK inhibitor U0126 (10 μM) promoted the inhibitory effect of TEN in HCC cells, indicating that although the ROS–ERK axis is likely to be a crucial axis in the cancer cell phenotype, this is not the only relevant pathway (Figures 7G–K). Overall, these results indicate that TEN inhibits the biological behavior of HCC cells through a ROS/ERK/JNK signaling axis.

### Downregulation of SIRT5 expression by TEN inhibits succinylation in Hep3B and HCCLM3 cells

**FIGURE 8:**
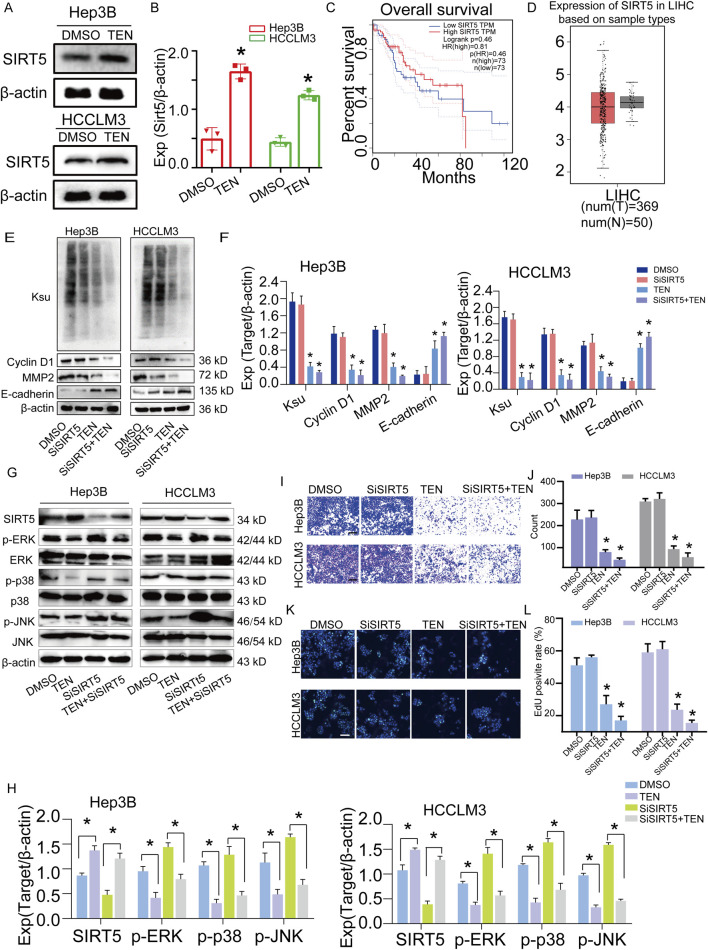
Downregulation of SIRT5 expression by TEN inhibits the succinylation level in Hep3B and HCCLM3 cells. **(A,B)** The protein expression of SIRT5 in Hep3B and HCCLM3 cells treated with TEN (20 μM) for 48 h. n = 3. **(C,D)** Survival analysis of SIRT5 in clinical samples of HCC and the expression. **(E,F)** Protein expression of Ksu, Cyclin D1, MMP2, and E-cadherin after downregulation of SIRT5 and treated with TEN (20 μM) in Hep3B and HCCLM3 cells. n = 3. **(G,H)** Western blotting analysis for detecting SIRT5, p-ERK, and p-p38, along with p-JNK at the protein level with TEN or SiSIRT5. n = 3. **(I,J)** The migration of cells after treated with SiSIRT5 and TEN (20 μM). n = 5. **(K, L)** The proliferation of cells after treated with SiSIRT5 and TEN (20 μM). n = 5. ANOVA was employed for multiple comparisons, whereas *t* tests were used for two‐group comparisons. *p < 0.05 indicates significant differences compared with the control group.

SIRT5 is an important desuccinylase; therefore, we investigated whether the downregulation of succinylation following TEN treatment was mediated by SIRT5 expression in HCC cells. This analysis found that SIRT5 expression was upregulated following TEN treatment (Figures 8A,B). Prognostic survival analysis also demonstrated that lower SIRT5 levels predicted a poorer prognosis in HCC patients and that its expression was lower in HCC clinical samples than in normal samples (Figures 8C,D). Furthermore, succinylation levels were increased after SIRT5 inhibition, while TEN treatment led to downregulated succinylation levels. Similarly, the patterns of cyclin D1, MMP2, and E-cadherin were similar (Figures 8E,F), while the findings for p-ERK/p-JNK with SiSIRT5 were similar regarding succinylation changes (Figures 8G,H). Next, SIRT5 overexpression provides further evidence to support the inhibitory effect of SIRT5 on p-ERK/p-JNK and succinylation (Supplementary Figure S3). In addition, HCC cell migration was increased after SIRT5 inhibition, and TEN treatment could further inhibit this effect (Figures 8I,J). Similarly, HCC cell proliferation was increased after SIRT5 inhibition, and TEN treatment could further inhibit this effect (Figures 8K,L). These results suggested that TEN regulates succinylation via SIRT5 by decreasing succinate levels in HCC cells. TEN increased the SIRT5 expression level, which further simultaneously inhibited the ERK/JNK signaling pathway in HCC cells.

### TEN treatment inhibits tumor growth *in vivo*

**FIGURE 9:**
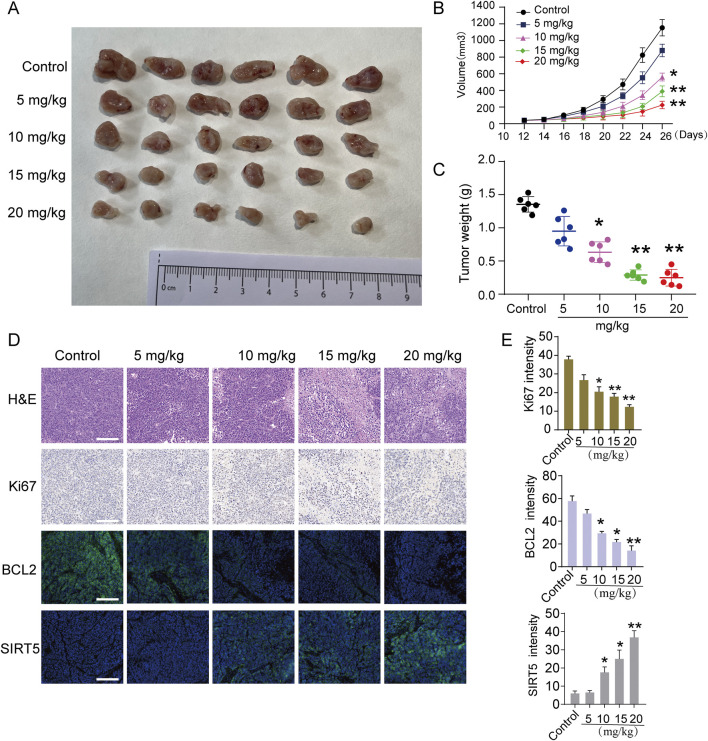
TEN suppressed transplanted tumor growth *in vivo*. Thirty nude mice were randomly divided into the negative control and TEN groups (5, 10, 15, and 20 mg/kg), injected once 2 days. n = 6. **(A)** Photograph of tumors from indicated mice. **(B)** Tumor volume of indicated mice. **(C)** Tumor weight of indicated mice. **(D)** H&E and IHC of Ki67, BCL2, and SIRT5 in indicated tumors. **(E)** Quantitative image analysis of Ki67, BCL2, and SIRT5 in **(D)**. Scale bar = 100 μm. ANOVA was employed for multiple comparisons, whereas *t* tests were used for two‐group comparisons. *p < 0.05 and * *p < 0.01 indicate significant differences compared with the control group.

To further confirm the anti-tumorigenic effect of TEN, a nude xenograft mouse model was generated by injecting Hep3B cells into one side of the axilla of nude mice, followed by treatment with TEN or the vehicle control. Following seven doses of TEN, the tumor weight and volume were significantly decreased compared to the control group, corroborating the *in vitro* anti-tumor effect (Figures 9A–C). To validate our results, we next performed H&E staining, Ki67 immunohistochemistry staining, and immunofluorescence staining on these tissues, as shown in Figures 9D,E. Here, TEN treatment significantly reduced the total number of Hep3B cells, while the expressions of Ki67 and BCL2 were highly inhibited, and the expression of SIRT5 was markedly upregulated; again, these aligned with the results of the *in vitro* experiments. Together, these results suggest that TEN exerts a significant anti-tumor effect *in vivo*.

## Discussion

HCC is one of the deadliest diseases and the leading cancers worldwide (Magyar et al., 2025). Typically, the pathogenesis of HCC is complex, and associated risk factors include alcoholism, steatohepatitis, and hepatitis B and C, which are contributors to an increasing incidence of HCC (Toh et al., 2023). The biological process of HCC is complex, and the onset is insidious; since currently available clinical diagnostic methods have certain limitations and the traditional therapies do not yield satisfactory results, the cure rate of HCC is very low, and fewer than one-third of patients with liver cancer can receive radical treatment (Jiang et al., 2023; Wang and Deng, 2023). In early-stage HCC patients, surgical resection and radiofrequency ablation are commonly used treatment approaches, but the results tend not to be satisfactory (Bosi et al., 2023). The discovery and development of novel therapeutic drugs are essential for the treatment of HCC.

Clinical chemotherapy and targeted therapy for HCC patients are usually associated with drug toxicity, especially in elderly and infirm patients. CDDP is a commonly used example with good efficacy, but it is usually associated with serious side effects, including toxicity to heart and kidney cells, along with drug resistance (Fang et al., 2024; Osman et al., 2023). This has led researchers to focus on safe and effective alternatives, such as extracts from traditional Chinese herbs. Research has shown that a variety of natural compounds extracted from plants have strong therapeutic effects on various diseases (Wang et al., 2024; Guo et al., 2023); for example, some extracts have shown positive anti-inflammatory and anticancer activities, which could reduce the influence of toxic side effects of chemotherapy drugs, in addition to strong therapeutic efficacy and safety profiles (Yang et al., 2023; Lan et al., 2022). Moreover, multiple studies on natural plant extracts for the treatment of HCC have demonstrated positive therapeutic effects, thereby supporting these approaches as important means of prolonging the survival of HCC patients and as targeted therapies for HCC (Pan et al., 2022; Xu et al., 2023). TEN, also known as senegenin, is an active compound extracted from the traditional Chinese herb *P. tenuifolia Willd.* Various studies have showed that TEN has many pharmacological effects, such as anti-inflammatory, antioxidant, and cognitive-enhancing properties (Tian et al., 2022). In this study, we explored the effect of TEN on HCC and explained its molecular mechanism. Our *in vitro* results show that TEN inhibits the proliferation of HCC cells by decreasing the proportion of cells entering the S-phase of the cell cycle, in parallel with reduced expression of proliferation-related proteins BCL2, CDK2, CDK4, CDK6, and cyclin D1. Furthermore, TEN inhibits the migration of HCC cells by reducing the expression of migration-related proteins N-cadherin, vimentin, and MMP2 while increasing the expression of E-cadherin. In addition, TEN induced apoptosis of HCC cells by modulating the expression of apoptosis regulatory proteins, such as caspase 8, MCL1, and survivin. Moreover, TEN effectively inhibits tumor growth *in vivo* by reducing the expression of Ki67 and BCL2. In addition, the highest concentration of TEN tested (50 µM) exhibited a stronger inhibitory effect on the proliferation and migration of HCC cells than the positive control CDDP-treated group, thereby supporting the excellent anti-HCC effects of TEN and its candidacy as an effective therapeutic drug.

Abnormal metabolic reprogramming is a hallmark of cancer (Xiao et al., 2023). Metabolic deregulation within cancer cells forms a microenvironment supportive of tumor growth, which leads to the enhancement of tumorigenic processes, including tumor cell proliferation, migration, and angiogenesis (You et al., 2023). Abnormally elevated arginine in tumor cells is a known component of metabolic reprogramming that alters glucose, amino acid, and nucleotide metabolism, thereby facilitating tumor development (Mossmann et al., 2023). In addition, glutamine can influence the TCA by acting as a carbon source through which it modulates the fate of tumor cells (Jin et al., 2023).

Our transcriptomic data analysis showed that there was a large amount of DEGs in HCC cells after TEN treatment, particularly in genes involved in the cell cycle, HCC, fructose metabolism, and mannose metabolism pathways. The LC–MS data showed that multiple metabolic pathways were enriched in the metabolome after TEN treatment, including central carbon metabolism in cancer, which comprises glycolysis, the TCA cycle, oxidative phosphorylation, and fatty acid metabolism. These metabolic processes are mainly involved in substance synthesis and energy metabolism and are known to be relevant to cancer cell metabolic reprogramming (Sun et al., 2023). Notably, we found that TEN-treated cells exhibited decreases in s-malate, succinate, and D-(−)salicin, indicating that TEN treatment significantly deregulates the TCA cycle and glycolytic metabolism in HCC cells, along with phenotypic processes including proliferation, migration, and apoptosis.

Succinate is one of the important intermediate metabolites in the TCA cycle of the mitochondrial matrix. Normally, succinate is the substrate for succinate dehydrogenase (SDH). Recent studies have emphasized the role of succinate in tumorigenicity, highlighting succinate as a metabolic product of tumor cells and emphasizing that SDH plays a tumor-suppressive role during oncogenesis (Dalla Pozza et al., 2020). Some studies have shown that succinate further enhances tumor cell migration by activating succinate receptor-1 (SUCNR-1) (Kuo et al., 2022). Furthermore, succinate has been shown to post-translationally modify protein succinylation; studies found that this effect is indirectly influenced through succinyl coenzyme A (Mills and O'Neill, 2014; Yu et al., 2025). In this study, we found that succinate levels decreased in TEN-treated HCC cells, which further caused a reduction in succinylation modification. Lysine succinylation is a novel PTM that regulates protein function and occurs in different cancers, and it can be regulated by enzymes such as SIRT5 and SIRT7 (Shen et al., 2023). It has been reported that SIRT5 is a known NAD^+^-dependent desuccinylase; it can directly catalyze the removal of succinyl groups from the lysine residues of the substrate protein SIRT5, and its expression is downregulated in liver cancer (Chen et al., 2018; Wu et al., 2024). SIRT5-mediated succinylation has been increasingly recognized as an important regulatory mechanism in liver physiology and pathology (Yue et al., 2026). Our study found that TEN increased SIRT5 expression, further decreasing succinylation in HCC cells and efficiently inhibiting tumorigenesis. In addition, our data unequivocally demonstrate that TEN regulates the succinylation modification of H3 at the H3K122su site in HCC cells. Previous studies on SIRT5 in HCC mainly focused on metabolic enzymes acting as substrates (Ji et al., 2024); however, in our study, we found that TEN regulates cellular biological behaviors by increasing the ROS content of HCC cells. Cellular ROS accumulation can lead to the activation of multiple cellular signaling pathways, including the AMPK, PI3K, and ERK signaling pathways (Liu et al., 2022; Yuan et al., 2022; Sun et al., 2024). Our results showed that TEN treatment significantly inhibited ERK, p38, and JNK protein phosphorylation, thereby suppressing cell proliferation and migration, and that this regulatory effect was achieved through the regulation of SIRT5. Unlike studies showing SIRT5 degradation by natural compounds in melanoma (Ji et al., 2024), we demonstrate that TEN-mediated SIRT5 activation exerts anti-tumor effects. Therefore, our research identifies for the first time that the TEN–SIRT5 axis regulates both succinylation and ERK/JNK signaling, thereby regulating oncogenic behaviors in HCC cells, such as proliferation and migration. However, the downstream molecular mechanisms require further analysis.

Together, our results demonstrated that TEN shows a potent antitumor effect, and the mechanism was related to the deregulation of central carbon metabolism as a mediator of HCC cell metabolism. Results from the present study demonstrated that TEN regulated succinylation in HCC cells and modulated the ERK/JNK signaling pathway, thereby regulating the proliferation and migration of HCC cells. Furthermore, SIRT5 was identified as a target of TEN, leading to the regulation of succinylation and ERK signaling pathways, which could further modulate the occurrence and development of HCC. These findings support it as a promising therapeutic candidate against HCC; however, its clinical application still faces critical challenges. Future studies will need to evaluate the safety profile through long-term toxicity studies to avoid potential adverse effects. Moreover, it is worth exploring whether TEN can be combined with existing clinical chemotherapy drugs (such as CDDP, sorafenib, and lenvatinib) for the treatment of liver cancer.

## Data Availability

The datasets presented in this study can be found in online repositories. The names of the repository/repositories and accession number(s) can be found at: https://www.ncbi.nlm.nih.gov/, PRJNA1007232.
