# Supplementary figures and images for: Tenuigenin regulates succinylation via SIRT5 for suppressing the tumorigenicity of hepatocellular carcinoma cells
Source: Front Pharmacol. 2026 Apr 24;17:1756680. doi: 10.3389/fphar.2026.1756680 (PMC13152865; doi:10.3389/fphar.2026.1756680)

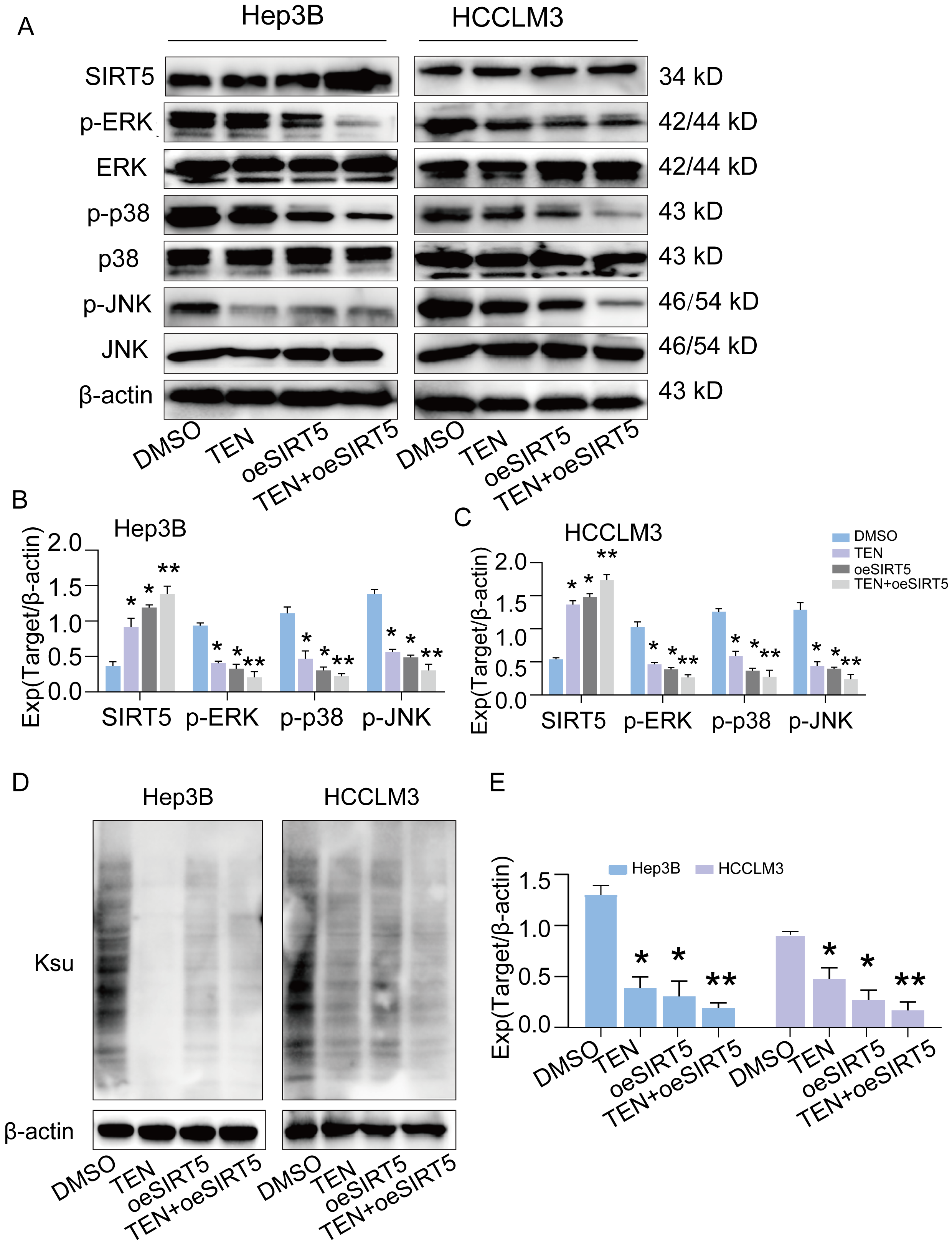

Supplement: Supplementary file 1 [file Image3.tif]

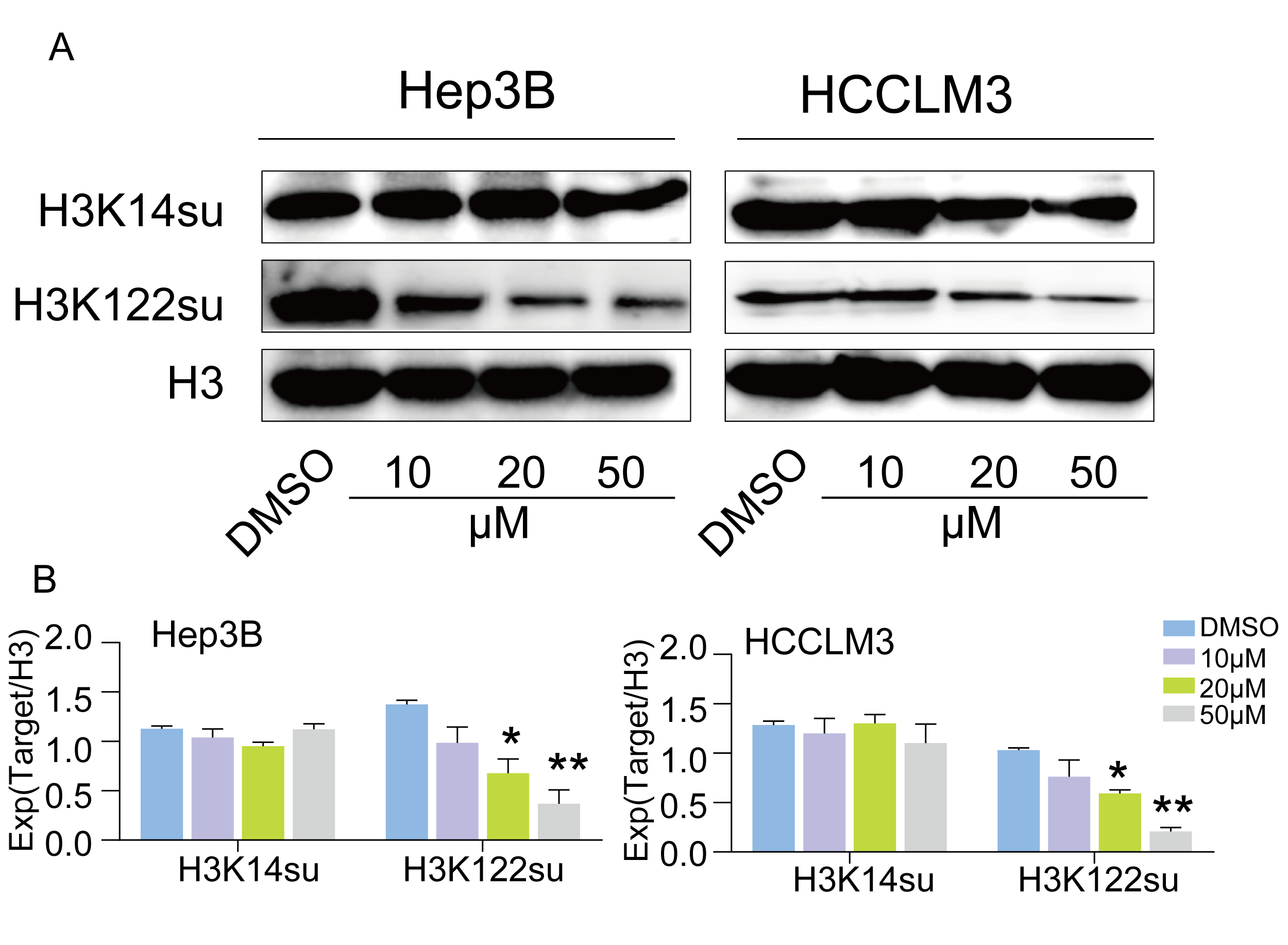

Supplement: Supplementary file 2 [file Image2.tif]

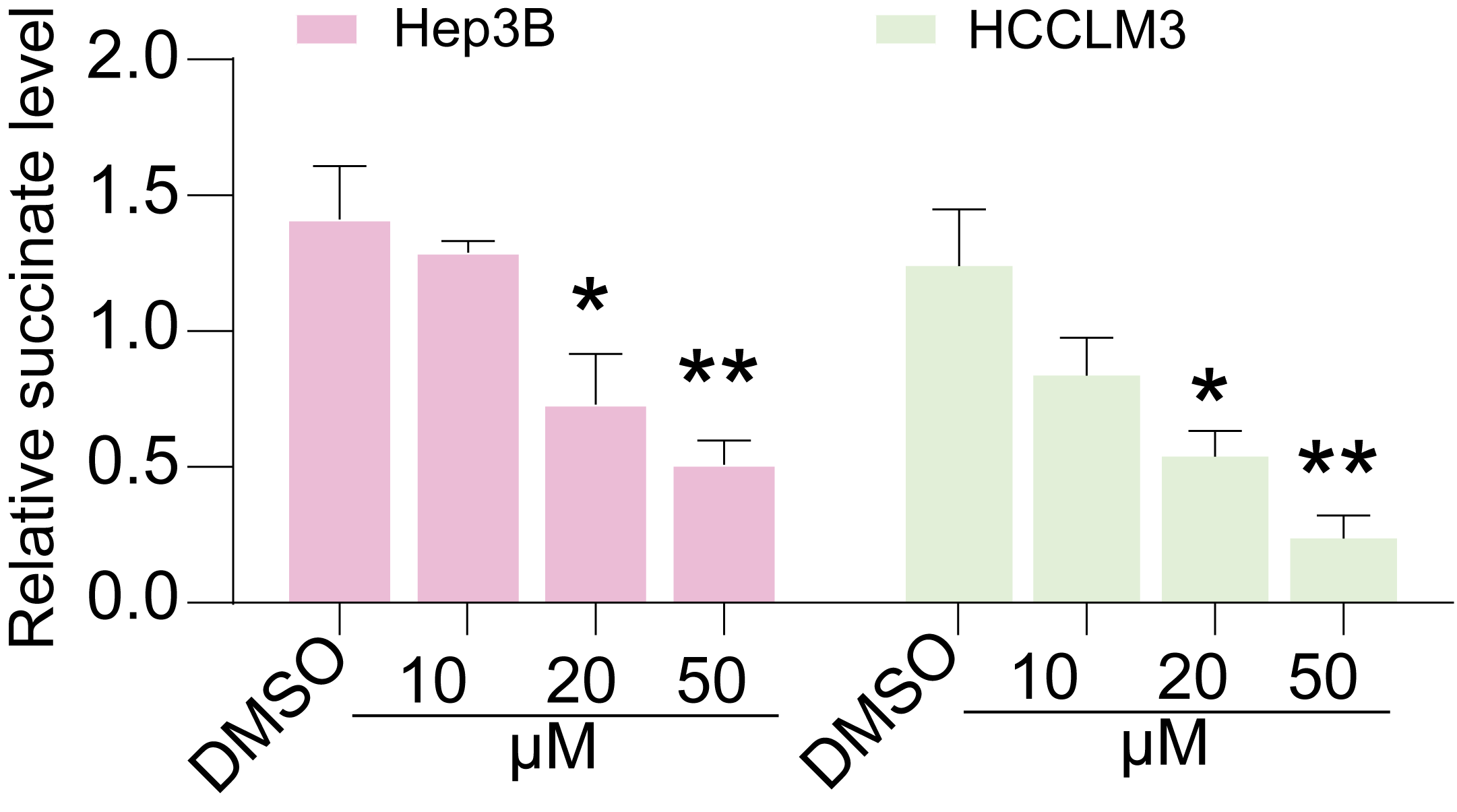

Supplement: Supplementary file 3 [file Image1.tif]
